# Hygiene in Its Relation to Therapeutics

**Published:** 1869-12

**Authors:** 


### Selections.

HYGIENE IN ITS RELATION TO THERAPEUTICS.—A paper read before the N. Y. Medical Journal Association, June 25th, 1869, By Alfred M. Carroll, M.D., etc., etc.

There are in this paper some facts that help to form the basis of a successful practice ; we select a few for our readers :

Lexicographers limit the department of hygiene to the prevention of disease, but etymology and experience show it has valid claims as a curative science. One legend of Mythology tells us Hygeia was the daughter of Esculapius. It is therefore probable she aided her father in the healing art. Plutarch says that while the vestibule of the Athenian Citadel was building under Pericles, one of the most skillful workmen fell, and was bruised so that his life was despaired of. Minerva told Pericles in a dream of a remedy, that speedily restored him ; in memory of which, he placed near the alter a brazen statute of the goddess; naming her Hygeia, or the Minerva of health.

Many maladies once thought to require the speedy interference of medical art, are now known to be self-limiting, naturally tending to a healthy end ; and not capable of being shortened or materially modified by the use of drugs ; it seems that all so-called acute diseases belong to this class.

The hygienic agents adapted to the treatment of disease, are good food, drink, and all other materials that will supply elements that are deficient in the organism.

Temperature, climate, clothing, exercise, bathing, electricity, sunlight, etc., may be used to benefit the system, and bring it to that healthy working condition necessary for the speedy and proper application of the nutritive elements taken as food.

Dr. Austin Flint, tells us certain parts of the body may suffer from the blood wanting the proper materials for their nutrition. Muscle is starved for want of nitrogen, the fats and animal heat for carbon, the brain for phosphorus, the blood for the salts of lime—potash, soda, iron and magnesia—so essential to a healthy state.

Most diseases are caused by defective–supply–assimilation, or by excessive waste of the elements essential to health, in the part diseased. All the elements required may be in the food taken, but the powers of assimilation being weak, one may starve to death on four meals a day.

For the intelligent use of alimentation, etc., I append the table formed from Barral's experiments on the human subject. Sulphur too (found in albuminoid matter) is required, and phosphorus (meats have much of it in soluble phosphates; it is also in most vegetable aliments), soda, potassa, magnesia, lime, iron, etc. If we can find out what

*Amount in grs. (Troy) furnished and eliminated during every twenty-four hours for each pound (avoirdupois) weight of the body.*

| | C. | H. | N. | O. | Water |
|---|---|---|---|---|---|
| Furnished by Alimination, | 53 90 | 8 39 | 4 20 | 49 00 | 294 73 |
| Furnished by Respiration, | | | | 156 12 | |
| | | | | 205 12 | |
| Eliminated by Skin and Lungs, | 49 42 | 7 62 | 2 17 | 202 60 | 121 18 |
| Eliminated by Kidneys, | 2 24 | 0 42 | 1 61 | 1 19 | 157 94 |
| Eliminated by Intestine, | 2 24 | 0 35 | 0 42 | 1 33 | 15 61 |
| | 53 90 | 8 39 | 4 20 | 205 12 | 294 73 |

elements are required; to supply them, a knowledge of the chemical composition alimentary matter is required.

Alimentary substances conducing to tissue formation, are called "plastic," and "respiratory" when they produce heat; the former comprising nitrogenized substances, the latter rich in hydrogen aud carbon. Vegetable food is richer in respiratory—animal food in nitrogenized—elements.

Under a deficiency of the hydro-carbons, nitrogenized substances may be converted to respiratory purposes,—indeed gelatin highly nitrogenized, serves no plastic end, but is purely calorific, and whether respiratory food may not be used to some extent for reparative purposes, is an unsettled point. The nervous tissue is doubtless nourished by fatty substances combining phosphorus from the mineral salts.

| | Plastic. | Respiratory. | | Plastic. | Respiratory. | | Plastic. | Resp'ry. |
|---|---|---|---|---|---|---|---|---|
| Rice | 7 55 | 90 45 | Corn | 12 30 | 80 60 | Barley | 12 96 | 79 19 |
| Wheat, f'm | 14 60 | 66 40 | Rye | 12 80 | 75 40 | Oats | 17 00 | 50 80 |
| Wheat, to | 17 20 | 64 60 | Peas | 23 40 | 50 00 | Beans | 24 00 | 48 50 |
| Potatoes | 1 40 | 19 30 | Cocoa | 18 00 | 67 60 | Chocolate | 13 00 | 67 60 |

Meats contain:

| | Fibrin and Albumen. | Gelatin. | Fatty matter. |
|---|---|---|---|
| Veal | 9 | 7 5 | 16 5 |
| Beef | 8 | 7 | 20 |
| Mutton | 5 5 | 7 | 40 |
| Pork | 4 5 | 5 5 | 50 |

Fish, according to species, contain: of nitrogenized matter, (including gelatin), from 13 to 24 per cent.; of fatty matter, from 6 36 to 13 per cent

We should note the comparative powers of gastric and intestinal dejection, and give as much food as can be digested and assimilated. Respiratory food may be necessary, and yet the alkaline intestiral digestive function inadequate, while the stomach is still in working order. Here, and in all cases where emaciation and innutrition are caused by excessive oxidation of albuminoid matter, gelatin will be useful since it offers respiratory materials digestible by the stomach.

If nervous power be defective, meats (rich in phosphates) or still better, fish should be largely taken. Meats, and milk too contain about one per cent. of iron, therefore they should be the chief diet in anæmic conditions.

This mode of treatment is strikingly shown in scurvy and some other diseases.

The particular desires of the patient should be observed; for specific wants of certain tissues, are often shown by a craving for certain kinds of food. In disease a strong desire for peculiar articles of diet, may be the still small voice of some suffering tissue, urging its wants above those of its fellows. The habits, and perversions of appetite have also to be considered.

Of accessory food; tea, coffee, tobbacco and alcohol, have besides any special qualities, the common property of lessening the disentegration and absorption of albuminous tissues, and may be useful in lessening any excessive waste in this direction.

The use of iron in anæmic, or the phosphates of hypophosphates in nervous asthenia is essentially hygienic—applying assimilably the alimentary principles wanting in the tissues. Cod liver oil is food, not physic, and other successful drugs will doubtless, as our knowledge advances, be termed aliments. If, as Dr. Dupre concludes, animal quinoidine be a normal constituent of the body, promoting fluorescence of tissues, be correct, then quinine may be placed on this list. Rice and arrow-root are thought to produce constipation, but this apparent result is caused by their leaving little excrement; they are therefore useful when rest for the lower bowel is wanted.

In cool weather a fire supplied with pure air from an inlet at the upperpart of the room, will offer the best means of ventilation. Under all circumstances, at least a thousand feet of air should be allowed per hour to each person in the apartment. The organic matter exhaled from the body is more hurtful than carbonic acid.

Dry air being a bad conductor of heat, in a dry, cool atmosphere the temperature of the body is lowered chiefly by radiation. On the respiratory organs dry air is directly cooling, owing to its affinity for moisture—which varies greatly with the temperature: a hundred cubic feet of air at 4°, is saturated with 6.6 grs. of water, but at 104° there are 216 grs. required.

Cold, dry air by its non-conducting property, conserves animal heat, but hot dry air by promoting evaporation of the perspiration produces a cooling effect; *vice versa*, a cold humid atmosphere is a speedy refrigerant, and warm moist air augments the bodily heat. An important application of these principles is seen in the use of vapor and hot air baths. The super-saturation of the vapor bath prevents evaporation of the perspiration, and the body accumulates caloric, but the bath of hot air with its large hydrometric capacity accelerates evaporation from the skin and lungs, abstracting a large amount of heat, hence it can be borne better and longer than the former.

Increased atmospheric pressure, perhaps, accelerates the circulation mechanically, and compressed air—having more oxygen in a given bulk, is used empirically in some diseases. Dr. G. Von Liebig has deduced, from experiment with compressed air, that when the subject becomes accustomed to breathing the denser medium, there is not any material difference in the number of respirations, the quantity of air respired, nor in the amount of carbonic acid eliminated per minute. Rarified air rendering greater the relative weight of the body, causes a tendency to congestion and hemorrhage, augments insensible perspiration, forcing the lungs to increased action, by furnishing less oxygen in a given volume, and thus quickens the circulation.

Artificially increasing the oxygen inspired, has in many cases produced gratifying results. Visceral engorgement or hæmorrhagic bowels require a warm, humid, and not too elevated a locality. Scrofulous, dropsical, diarrhœal disorders, and generally those with excessive mucous secretion call for warmth and dryness. Many nervous maladies require a temperate, moist climate. As dry, hot climates derange the liver and bowels, the opposite conditions relieve them.

Heat tends to increase the activity of nutrition, but the amount of caloric carried off by transpiration, by calling an excess of blood to the surface, induces sluggish digestion and rapid exhaustion. Bile and semen are increased in hot climates, the other secretions are lessened by abundant perspiration. As the skin may act vicariously for the kidneys, hot, dry climates or the hot air bath—if not contra-indicated, may be beneficial.

Cold diminishes cutaneous action, drives the blood from the surface to the internal organs, renders the circulation sluggish and increases the secretions generally. I protest against the American fondness for furnace and stove heat—without provision for increased evaporation of water for the increased hygrometric capacity of heated air, causing probably our national tendency to sore throat. Light greatly conduces to health, stimulating the nervous, and physical system. It causes wounds to heal more rapidly than when covered.—*Nashville Journal of Medicine and Surgery.*

